# Plasma Homocysteine Levels and Intracerebral Hemorrhage: A Mendelian Randomization Analysis

**DOI:** 10.7759/cureus.86453

**Published:** 2025-06-20

**Authors:** Shuheng Chen, Xiangli Guo, Yingchao He, Yinzhou Wang

**Affiliations:** 1 Department of Neurology, Fujian Provincial Hospital, Fuzhou, CHN; 2 Department of Medical Physics, Fujian Institute of Hematology, Fujian Medical University Union Hospital, Fuzhou, CHN; 3 Department of Neurology, Fujian Key Laboratory of Medical Analysis, Fujian Academy of Medical Sciences, Fuzhou, CHN

**Keywords:** genetic instrument variables, gwas, homocysteine, intracerebral hemorrhage, mendelian randomization

## Abstract

Background

Hyperhomocysteinemia is a risk factor for ischemic stroke, but its role in hemorrhagic stroke remains unclear. Elevated homocysteine (Hcy) levels may promote endothelial dysfunction, oxidative stress, and extracellular matrix degradation, all of which can contribute to vessel wall fragility and susceptibility to rupture. This study aimed to investigate the causal relationship between plasma homocysteine level and spontaneous intracerebral hemorrhage (ICH).

Method

We used Mendelian randomization (MR) analysis with data from two independent groups to determine whether plasma homocysteine levels have a causal relationship with ICH. Genetic variants linked to homocysteine levels were taken from the largest available genome-wide association study (GWAS), which included 44,147 people of European ancestry. Data on primary (non-traumatic) spontaneous intracerebral hemorrhage were obtained from the FinnGen database, which comprised 7,763 cases and 412,105 controls, all of European ancestry. Primary Mendelian randomization estimates were calculated using the inverse-variance weighted method, with additional analyses conducted using alternative methods and multiple sensitivity tests.

Result

We found that genetic predisposition to higher plasma homocysteine levels was significantly associated with an increased risk of spontaneous ICH. Specifically, each one-unit increase in the natural logarithm of genetically predicted homocysteine levels was associated with 41% higher odds of ICH (odds ratio [OR] = 1.41, 95% confidence interval [CI]: 1.11-1.80, P = 0.0046), based on the inverse-variance weighted (IVW) method. This Mendelian randomization analysis involved 13 independent genetic variants (SNPs) as instrumental variables. No significant heterogeneity was observed among these SNPs (Cochran’s Q test, P = 0.7818), indicating consistency across the genetic instruments used. Additionally, MR-Egger regression intercept analysis did not reveal evidence of horizontal pleiotropy (intercept = 0.0178, p = 0.3676), further supporting the robustness of our results. Visual inspection of scatter plots also did not identify obvious outliers or influential SNPs.

Conclusions

Total homocysteine levels were found to be associated with an increased risk of ICH. These findings suggest that homocysteine-lowering strategies may warrant further investigation as a potential approach to reduce the risk and progression of ICH, particularly in individuals with a genetic predisposition to elevated homocysteine levels.

## Introduction

Homocysteine (Hcy) is a sulfur-containing amino acid formed during the metabolism of methionine. Various factors can lead to its accumulation in the blood, resulting in hyperhomocysteinemia. A common cause of elevated homocysteine is vitamin B12 or folate deficiency, both of which impair the remethylation of homocysteine to methionine via the enzyme methionine synthase. Specifically, vitamin B12 acts as a cofactor, while folate provides the methyl group required for this reaction. In addition, genetic variants in 5,10-methylenetetrahydrofolate reductase (MTHFR) [1]. Additional factors that may elevate homocysteine levels include a methionine-rich diet, smoking, and a sedentary lifestyle [2]. Previous studies have shown a strong association between hyperhomocysteinemia and atherosclerosis, cardiovascular disease, and stroke [3-5]. Beyond its atherogenic role, elevated homocysteine may also contribute to hemorrhagic stroke by promoting endothelial dysfunction, oxidative stress, and vascular fragility.

Intracerebral hemorrhage (ICH) accounts for only about 15% of all strokes but is often fatal and disabling due to bleeding within or around the brain. According to the latest statistics of the American Heart Association (AHA) 2024 latest statistics, in 2021, stroke caused approximately 7.44 million deaths globally, with ICH responsible for 3.38 million of these deaths [6]. ICH is primarily associated with uncontrolled hypertension, diabetes mellitus (DM), and excessive alcohol consumption [7]. Although less common, the severity of ICH makes it a critical focus in medical research and treatment [8].

There is compelling evidence that hyperhomocysteinemia is a pathogenic risk factor for ischemic stroke [9]. The mechanisms by which hyperhomocysteinemia may lead to vascular diseases include thrombosis [10], increased production of hydrogen peroxide [11], endothelial dysfunction [12], and enhanced oxidation of low-density lipoprotein (LDL) [13]. This risk factor can be controlled by supplementation with folic acid and B vitamins, such as vitamin B6 and vitamin B12, which are key cofactors in homocysteine metabolism [14]. However, the association between hyperhomocysteinemia and ICH remains unclear. Randomized controlled trials (RCTs) are regarded as the “gold standard” for determining causality between interventions and outcomes, as random allocation helps mitigate biases from both known and unknown confounders. However, RCTs often encounter practical limitations such as high expenses, lengthy durations, and potential ethical concerns [15]. Mendelian randomization (MR), a genetic epidemiology method, uses genetic variants as tools to test whether modifiable risk factors or biological markers directly cause certain diseases. By mimicking the random assignment used in RCTs, Mendelian randomization helps reduce biases caused by confounding factors and reverse causation [16]. Since genotypes are established at birth, causal inferences of Mendelian randomization are minimally affected by such biases, serving as a valuable complement to the constraints of RCTs.

In this study, we conducted two-sample Mendelian randomization using summary statistics from large-scale genome-wide association studies (GWAS) of homocysteine levels and cerebral hemorrhage to assess the causal effect of homocysteine on the risk of ICH.

## Materials and methods

Study design

**Figure 1:**
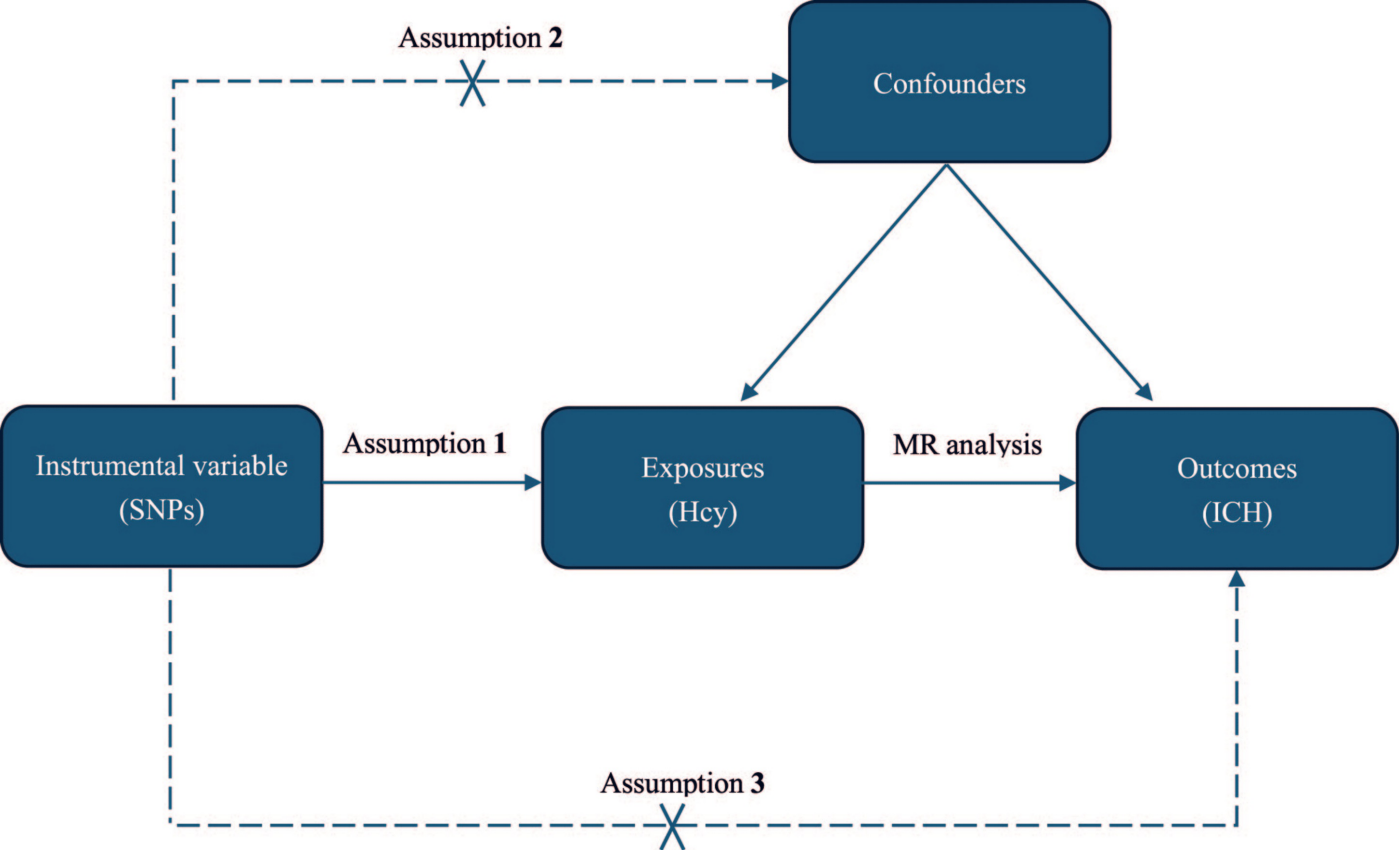
Three main assumptions of a MR study One: SNPs must be associated with Hcy; two: SNPs must be independent of confounders; and three: SNPs should not be directly associated with patient outcomes. MR: Mendelian randomization; SNP: single nucleotide polymorphism; Hcy: homocysteine; ICH: intracerebral hemorrhage. Image Credits: Shuheng Chen

To obtain reliable causal inferences, Mendelian randomization studies must meet three key assumptions: 1. Relevance: The instrumental variable (IV) must be strongly associated with the exposure of interest, meaning that it significantly influences exposure. 2. Independence: The instrumental variable should be independent of any confounding factors, ensuring that it is not associated with other variables that could confound the relationship between exposure and outcome. 3. Exclusion Restriction: The instrumental variable should affect the outcome only through exposure and not through any other pathway [16,17]. This means that the effect of the instrumental variable on the outcome is entirely mediated by exposure (Figure 1).

Data sources

The instrumental variables related to plasma homocysteine levels were obtained from a genome-wide association study (GWAS) meta-analysis [18] conducted by van Meurs et al. This study combined data from nine different groups, involving a total of 44,147 participants of European ancestry, and identified 18 single nucleotide polymorphisms (SNPs) significantly associated with plasma homocysteine levels (Table 1). FinnGen is an ongoing project dedicated to advancing human health and facilitating the identification of therapeutic targets through genetic research [19]. We used the 11th release of the FinnGen GWAS summary statistics, which included data on non-traumatic intracranial hemorrhage from 7,763 cases and 412,105 controls of European descent. The FinnGen study obtained ethical approval, and all participants provided written informed consent.

**Table 1:** Characteristics of SNPs for homocysteine SNP: single nucleotide polymorphism; EA: effect allele; OA: other allele; EAF: effect allele frequency; β: per allele effect on exposures; SE: standard error; p: p-value for the genetic association.

SNP	Chromosome	Nearest gene	EA	OA	EAF	β	SE	p
rs12134663	1	MTHFR	A	C	0.8	-0.101	0.011	2.54×10^-21^
rs12780845	10	CUBN	A	G	0.65	0.0529	0.009	7.80×10^-10^
rs12921383	16	DPEP1/FANCA	T	C	0.87	-0.09	0.014	8.22×10^-11^
rs154657	16	DPEP1	A	G	0.47	0.0963	0.007	1.74×10^-43^
rs1801133	1	MTHFR	A	G	0.34	0.1583	0.007	4.34×10^-104^
rs1801222	10	CUBN	A	G	0.34	0.0453	0.007	8.43×10^-10^
rs2251468	12	HNF1A-AS1	A	C	0.65	-0.0512	0.007	1.28×10^-12^
rs2275565	1	MTR	T	G	0.21	-0.0542	0.009	1.96×10^-10^
rs234709	21	CBS	T	C	0.45	-0.0718	0.007	3.90×10^-24^
rs2851391	21	CBS	T	C	0.47	0.056	0.008	1.70×10^-12^
rs42648	7	GTPB10	A	G	0.4	-0.0395	0.007	1.97×10^-08^
rs4660306	1	MMACHC	T	C	0.33	0.0435	0.007	2.33×10^-09^
rs548987	6	SLC17A3	C	G	0.13	0.0597	0.01	1.12×10^-08^
rs7130284	11	NOX4	T	C	0.07	-0.1242	0.013	1.88×10^-20^
rs7422339	2	CPS1	A	C	0.33	0.0864	0.008	4.58×10^-27^
rs838133	19	FUT2	A	G	0.45	0.0422	0.007	7.48×10^-09^
rs9369898	6	MUT	A	G	0.62	0.0449	0.007	2.17×10^-10^
rs957140	11	NOX4	A	G	0.45	-0.045	0.008	2.43×10^-08^

Instrumental variables extraction

To meet the relevance criterion of Assumption 1, we selected independent SNPs (R² < 0.001, with a 10,000 kb distance threshold) from the original set of 18 SNPs that showed a genome-wide significant association with plasma homocysteine levels (p < 5 × 10⁻⁸). These SNPs were chosen as potential instrumental variables (IVs). Additionally, to ensure that the instrumental variables had sufficient power to detect a causal effect of the exposure on the outcome, we calculated the F-statistics using an online tool [31]. SNPs with an F-statistic above 10 were considered strong enough to provide reliable estimates and were selected as valid instrumental variable candidates. Potential confounding factors were assessed using PhenoScanner (Cambridge, UK) to exclude instrument variants with significant associations with the outcome, and none of the SNPs were removed.

Mendelian randomization analysis

To evaluate whether plasma homocysteine levels have a causal effect on ICH, we primarily used the inverse-variance weighted Mendelian randomization (IVW-MR) method. This method combines the effect estimates from individual SNPs through a meta-analysis, producing an overall measure of how exposure (homocysteine) influences the outcome (ICH). Specifically, we applied a random-effects model when there was significant variation (heterogeneity) among the SNP results (p < 0.05). If there was no significant heterogeneity, we used a fixed-effects model instead. We also performed additional analyses using methods such as MR-Egger, weighted median, simple mode, and weighted mode to check and strengthen the main IVW-MR findings. Statistical significance was defined as a two-sided p-value < 0.05. All analyses were done using R software (version 4.3.2) and the TwoSampleMR package.

Sensitivity analysis

To ensure the reliability of our Mendelian randomization results, we conducted several sensitivity analyses, including testing for heterogeneity using Cochran's Q statistic from the IVW and MR-Egger methods, assessing potential horizontal pleiotropy through the MR-Egger intercept test, and performing a leave-one-out analysis to determine whether any individual SNP had an outsized impact on the overall causal estimate.

## Results

Genetic instrument variables

To handle linkage disequilibrium (LD), we excluded 5 out of the initial 18 SNPs. The remaining 13 SNPs were used as instrumental variables in our study (Table 2). Among these, eight SNPs (rs9369898, rs1801133, rs154657, rs4660306, rs548987, rs1801222, rs838133, and rs12780845) were genetically linked to higher plasma homocysteine levels, while five SNPs (rs2275565, rs2251468, rs234709, rs7130284, and rs42648) were associated with genetically predicted lower homocysteine levels. All selected SNPs had strong statistical power, indicated by an F-statistic greater than 10.

**Table 2:** The characteristics of 13 SNPs and their associations with homocysteine SNP: single nucleotide polymorphism; EA: effect allele; OA: other allele; EAF: effect allele frequency; β: beta coefficient for effect allele; SE: standard error.

SNP	EA	OA	EAF	β	SE	P-value
rs12780845	A	G	0.65	0.0529	0.009	7.80×10^-10^
rs154657	A	G	0.47	0.0963	0.007	1.74×10^-43^
rs1801133	A	G	0.34	0.1583	0.007	4.34×10^-104^
rs1801222	A	G	0.34	0.0453	0.007	8.43×10^-10^
rs2251468	A	C	0.65	-0.0512	0.007	1.28×10^-12^
rs2275565	T	G	0.21	-0.0542	0.009	1.96×10^-10^
rs234709	T	C	0.45	-0.0718	0.007	3.90×10^-24^
rs42648	A	G	0.40	-0.0395	0.007	1.97×10^-08^
rs4660306	T	C	0.33	0.0435	0.007	2.33×10^-09^
rs548987	C	G	0.13	0.0597	0.01	1.12×10^-08^
rs7130284	T	C	0.07	-0.1242	0.013	1.88×10^-20^
rs838133	A	G	0.45	0.0422	0.007	7.48×10^-09^
rs9369898	A	G	0.62	0.0449	0.007	2.17×10^-10^

The causal association between homocysteine and ICH

**Figure 2:**
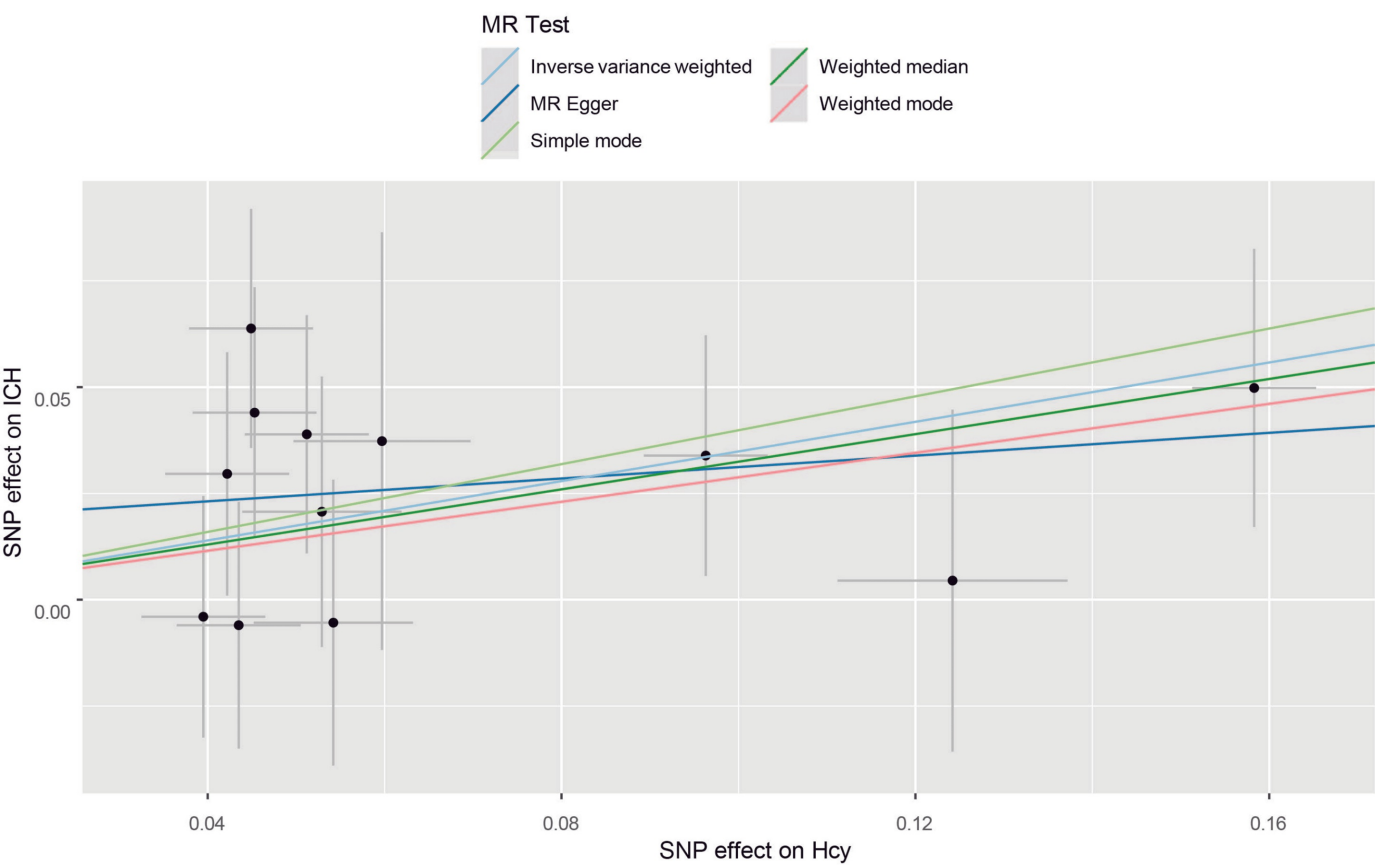
The scatter plot from genetically predicted homocysteine on ICH The X-axis represents the impact of SNPs on homocysteine, the Y-axis represents the impact of SNPs on ICH, and each black dot indicates an SNP, plotted by the estimate of SNP on plasma homocysteine level and the estimate of SNP on the risk of ICH with standard error bars. The slopes of the lines correspond to the causal estimates using each method.

**Figure 3:**
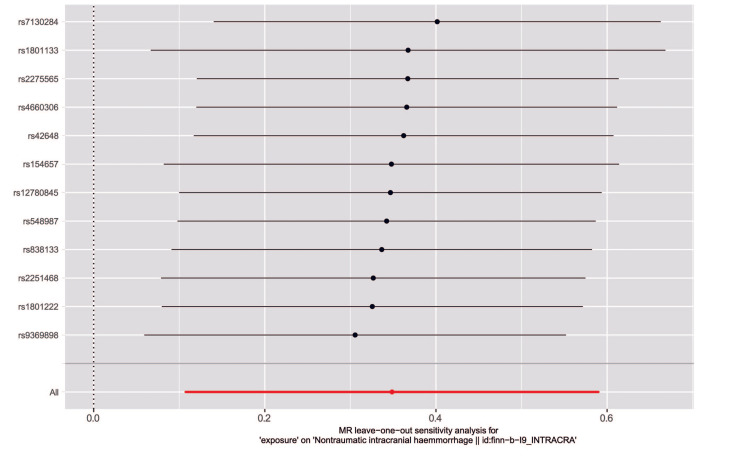
The results of leave-one-out methods for sensitivity analysis X-axis: Estimated causal effects of the remaining SNPs after excluding one.

Inverse-variance weighted (IVW) analysis indicated that genetically elevated homocysteine levels were associated with higher odds of ICH. The odds ratio (OR) for ICH per 1 SD increase in genetically predicted homocysteine was 1.41 (95% CI, 1.11-1.80; p = 0.0046) (Table 3). Although some methods do not produce statistically significant findings, consistency in the causal direction is noteworthy. With p-values for inverse-variance weighted and weighted median both below 0.05, these results support confidence in the Mendelian randomization analysis, confirming the association between homocysteine and ICH. The scatter plot showed a positive correlation between homocysteine levels and ICH (Figure 2). The p-values of Q statistics for IVW and MR-Egger analyses indicated no evidence of heterogeneity (MR-Egger: Q statistic = 6.3195, p = 0.7877; IVW: Q statistic = 7.2099, p = 0.7818). The MR-Egger intercept suggested no horizontal pleiotropy with a p-value > 0.05) (Table 4). Leave-one-out analysis showed that the overall estimate remained stable regardless of the exclusion of individual SNPs (Figure 3). These Mendelian randomization analysis results indicated that genetically predicted elevated plasma homocysteine levels are positively associated with ICH risk.

**Table 3:** MR estimates from each method of assessing the causal effect of homocysteine on the risk of ICH MR: Mendelian randomization; SE: standard error; OR: odds ratio; p < 0.05 was considered statistically significant.

MR Method	SE	OR (95% CI)	P-value
MR Egger	0.2584	1.144 (0.689, 1.898)	0.6147
Weighted median	0.1654	1.383 (1.000, 1.913)	0.0498
Inverse variance weighted	0.1231	1.417 (1.113, 1.804)	0.0046
Simple mode	0.2444	1.490 (0.923, 2.405)	0.1311
Weighted mode	0.1751	1.334 (0.946, 1.880)	0.1284

**Table 4:** MR-Egger regression intercept Hcy: homocysteine; ICH: intracerebral hemorrhage; p < 0.05 was considered statistically significant.

Exposure	Outcome	Egger_intercept	p-value
Hcy	ICH	0.01776826	0.3676274

## Discussion

In this study, we utilized large-scale GWAS data and Mendelian randomization for the first time to investigate the potential causal relationship between genetically predicted homocysteine levels and ICH. As expected, the genetically predicted plasma homocysteine levels were positively associated with ICH risk. This finding is consistent with the results of the meta-analysis conducted by Zhou et al., which included seven studies involving 667 ICH patients, 1821 ischemic stroke patients, and 2500 healthy controls and revealed that plasma homocysteine levels were significantly elevated in ICH patients compared to healthy controls [20].

Hypertension is the leading cause of spontaneous ICH, primarily by inducing microaneurysm formation at arteriolar bifurcations. Multiple studies have identified a strong association between specific apolipoprotein E (APOE) alleles and increased susceptibility to ICH. In particular, the ɛ4 and ɛ2 alleles are linked to cerebral amyloid angiopathy and an elevated risk of lobar hemorrhages [21,22]. Additionally, a study on Alzheimer’s disease (AD) suggested that carriers of the APOE ε4 allele exhibit higher levels of oxidative stress and decreased antioxidant activity in the hippocampal region of the brain. These findings suggest that the APOE ε4 allele may contribute to endothelial damage and increase the risk of ICH [23]. It is reasonable to believe that the synergistic effect of the APOE ε4 allele and homocysteine exacerbates endothelial damage and contributes to ICH occurrence. Polymorphisms in the methylenetetrahydrofolate reductase (MTHFR) gene have also been associated with ICH. MTHFR is a critical enzyme in folate metabolism that regulates one-carbon units essential for protein synthesis and facilitates the metabolism of homocysteine using folate. A common genetic variant, MTHFR C677T (rs1801133), with a T allele frequency of approximately 10% in populations of European ancestry, significantly reduced enzyme efficiency, resulting in elevated plasma homocysteine levels. Experimental studies have demonstrated that elevated homocysteine levels can induce changes in the blood vessel wall [24] through mechanisms involving oxidative stress and pro-inflammatory responses, contributing to endothelial injury [25]. Oxidative and nitrosative stresses are key contributors to the vascular effects of hyperhomocysteinemia. These stressors diminish NO bioavailability, exacerbate endothelial dysfunction, and impair cerebrovascular function [26]. Experimental research in both animal and human models suggests that oxidative stress, potentially triggered by hyperhomocysteinemia, disrupts endothelium-dependent nitric oxide signaling, further promoting vascular damage and dysfunction [27,28].

Clinical studies have shown that supplementation with folic acid and vitamin B12 lowers homocysteine concentration by approximately 3 μmol/L [29]. A multicenter randomized controlled trial demonstrated that folic acid supplementation can reduce the risk of ischemic stroke in individuals with hypertension but has no significant effect on the prevention of hemorrhagic stroke [30]. Therefore, the benefits of folic acid supplementation in the general population require further clinical investigation.

Our study has several strengths. First, by using genetic variants randomly allocated at conception, our analysis avoids common problems seen in traditional observational studies, such as residual confounding and reverse causation. This means our findings suggesting a causal relationship between homocysteine levels and ICH are less likely to be biased. Second, our study employed Mendelian randomization, using large-scale genetic data from GWAS, to strengthen the reliability of our conclusions. We specifically chose GWAS data from European populations to minimize biases from population differences. However, several limitations should be acknowledged. Our analysis focused solely on genetic determinants of homocysteine levels and did not account for environmental or lifestyle factors, such as diet, vitamin status, renal function, and smoking. These factors may modify the relationship between homocysteine and ICH. The lack of gene-environment interaction data may limit the completeness of our findings and reduce their translational relevance. In addition, the exclusive use of European-ancestry datasets may restrict the generalizability of our results to other ethnic populations, since genetic architecture and environmental exposures can differ across populations. Finally, Mendelian randomization analyses rely on specific assumptions, including the absence of horizontal pleiotropy and correct model specification. Although we conducted sensitivity analyses to assess these assumptions, they cannot be definitively proven. Therefore, although our genetic findings support causal inference, further validation through mechanistic studies, functional assays, and interventional trials will be necessary before the results can be considered ready for clinical application.

## Conclusions

In conclusion, our Mendelian randomization analysis provided clear and robust evidence that genetically elevated homocysteine levels are causally linked to an increased risk of spontaneous intracerebral hemorrhage. By utilizing genetic data, this study minimized typical observational study biases, strengthening the argument that homocysteine may serve as a modifiable risk factor for spontaneous intracerebral hemorrhage. However, our findings also highlight the necessity for further investigation. Future studies, particularly large-scale randomized controlled trials and detailed biological research are needed to confirm these results and to better understand how homocysteine influences spontaneous intracerebral hemorrhage development. Ultimately, such studies could inform new approaches to prevention and treatment, potentially reducing the burden of this severe neurological condition.

## References

[1] Oliveira IO, Silva LP, Borges MC (2017). Interactions between lifestyle and MTHFR polymorphisms on homocysteine concentrations in young adults belonging to the 1982 Pelotas Birth Cohort. Eur J Clin Nutr.

[2] Dinç N, Yücel SB, Taneli F, Sayın MV (2016). The effect of the MTHFR C677T mutation on athletic performance and the homocysteine level of soccer players and sedentary individuals. J Hum Kinet.

[3] Homocysteine Studies Collaboration (2002). Homocysteine and risk of ischemic heart disease and stroke: a meta-analysis. JAMA.

[4] Esse R, Barroso M, Tavares de Almeida I, Castro R (2019). The contribution of homocysteine metabolism disruption to endothelial dysfunction: state-of-the-art. Int J Mol Sci.

[5] Nygård O, Nordrehaug JE, Refsum H (1997). Plasma homocysteine levels and mortality in patients with coronary artery disease. N Engl J Med.

[6] Martin SS, Aday AW, Almarzooq ZI (2024). 2024 heart disease and stroke statistics: a report of US and global data from the American Heart Association. Circulation.

[7] Boulouis G, Morotti A, Goldstein JN, Charidimou A (2017). Intensive blood pressure lowering in patients with acute intracerebral haemorrhage: clinical outcomes and haemorrhage expansion. Systematic review and meta-analysis of randomised trials. J Neurol Neurosurg Psychiatry.

[8] Thabet AM, Kottapally M, Hemphill JC 3rd (2017). Management of intracerebral hemorrhage. Handb Clin Neurol.

[9] Rabelo NN, Telles JP, Pipek LZ (2022). Homocysteine is associated with higher risks of ischemic stroke: A systematic review and meta-analysis. PLoS One.

[10] den Heijer M, Koster T, Blom HJ (1996). Hyperhomocysteinemia as a risk factor for deep-vein thrombosis. N Engl J Med.

[11] Stamler JS, Slivka A (1996). Biological chemistry of thiols in the vasculature and in vascular-related disease. Nutr Rev.

[12] Chambers JC, McGregor A, Jean-Marie J (1999). Demonstration of rapid onset vascular endothelial dysfunction after hyperhomocysteinemia: an effect reversible with vitamin C therapy. Circulation.

[13] Leerink CB, van Ham AD, Heeres A (1994). Sulfhydryl compounds influence immunoreactivity, structure and functional aspects of lipoprotein (a). Thromb Res.

[14] Clarke R, Halsey J, Lewington S (2010). Effects of lowering homocysteine levels with B vitamins on cardiovascular disease, cancer, and cause-specific mortality: Meta-analysis of 8 randomized trials involving 37 485 individuals. Arch Intern Med.

[15] Chan GC, Lim C, Sun T (2022). Causal inference with observational data in addiction research. Addiction.

[16] Davies NM, Holmes MV, Davey Smith G (2018). Reading Mendelian randomisation studies: a guide, glossary, and checklist for clinicians. BMJ.

[17] Emdin CA, Khera AV, Kathiresan S (2017). Mendelian randomization. JAMA.

[18] van Meurs JB, Pare G, Schwartz SM (2013). Common genetic loci influencing plasma homocysteine concentrations and their effect on risk of coronary artery disease. Am J Clin Nutr.

[19] Kurki MI, Karjalainen J, Palta P (2023). FinnGen provides genetic insights from a well-phenotyped isolated population. Nature.

[20] Zhou Z, Liang Y, Qu H (2018). Plasma homocysteine concentrations and risk of intracerebral hemorrhage: a systematic review and meta-analysis. Sci Rep.

[21] MCarron MO, Muir KW, Weir CJ (1998). The apolipoprotein E ε4 allele and outcome in cerebrovascular disease. Stroke.

[22] Woo D, Sauerbeck LR, Kissela BM (2002). Genetic and environmental risk factors for intracerebral hemorrhage: preliminary results of a population-based study. Stroke.

[23] Persson T, Lattanzio F, Calvo-Garrido J (2017). Apolipoprotein E4 elicits lysosomal cathepsin D release, decreased thioredoxin-1 levels, and apoptosis. J Alzheimers Dis.

[24] Faraci FM, Lentz SR (2004). Hyperhomocysteinemia, oxidative stress, and cerebral vascular dysfunction. Stroke.

[25] Johnson KL, Tiedeman T, Peterson H (2023). Potential mechanism for hyperhomocysteinemia in Greyhound dogs. J Vet Intern Med.

[26] De Silva TM, Miller AA (2016). Cerebral small vessel disease: targeting oxidative stress as a novel therapeutic strategy?. Front Pharmacol.

[27] Ungvari Z, Csiszar A, Edwards JG (2003). Increased superoxide production in coronary arteries in hyperhomocysteinemia: role of tumor necrosis factor-alpha, NAD(P)H oxidase, and inducible nitric oxide synthase. Arterioscler Thromb Vasc Biol.

[28] Eberhardt RT, Forgione MA, Cap A (2000). Endothelial dysfunction in a murine model of mild hyperhomocyst(e)inemia. J Clin Invest.

[29] Clarke R, Armitage J (2000). Vitamin supplements and cardiovascular risk: review of the randomized trials of homocysteine-lowering vitamin supplements. Semin Thromb Hemost.

[30] Huo Y, Li J, Qin X (2015). Efficacy of folic acid therapy in primary prevention of stroke among adults with hypertension in China: the CSPPT randomized clinical trial. JAMA.

[31] (2025). Shinyapps: Bias and Type 1 error rate for Mendelian randomization with sample overlap. http://sb452.shinyapps.io/overlap/.

